# Neurodiversity‐Affirming Principles in Oncology Treatment: A Lived‐Experience Case Study

**DOI:** 10.1002/pon.70394

**Published:** 2026-02-24

**Authors:** Rebecca Trevethick, Simon Baron‐Cohen, Elizabeth Weir, Carrie Allison, Matthew C. Fysh, David P. Laplante, Malini Dey, Jessica E. Opie

**Affiliations:** ^1^ no affiliation; ^2^ Autism Research Centre, Department of Psychiatry University of Cambridge Cambridge UK; ^3^ Centre for Child Development and Mental Health Lady Davis Institute Jewish General Hospital Montreal Quebec Canada; ^4^ Primary Care Unit, Department of Public Health and Primary Care University of Cambridge Cambridge UK

**Keywords:** attention‐deficit/hyperactivity disorder, autism (autism spectrum disorders), cancer, mental health, neurodivergent, oncology (general)

## Abstract

Cancer care, with its complex and variable protocols, frequent appointments, and high‐stress, may present additional challenges for neurodivergent patients, and suitable care accommodations are necessary. Yet, a dearth of literature and an associated understanding on neuro‐inclusive cancer care exists. The current case study aims to: (1) explore and identify barriers and challenges to neurodivergent cancer care; and (2) generate neuro‐inclusive oncology‐specific recommendations to guide healthcare providers in offering neuro‐affirming care. To do this, we present the first author's lived experience navigating stage IV breast cancer as a neurodivergent individual and psychologist, exploring barriers and challenges encountered. Her personal and professional experience uniquely positions her to offer insights into this intersection. Based on her experience, and complemented by published data, we generate recommendations for neuro‐inclusive care. Challenges and barriers identified include limited oncology provider knowledge, awareness, and tolerance of neurodivergence, and insufficient suitable accommodations to difficulties with standard care practices. Recommendations encompass six domains: patient communication, physical environments, physical pain, administrative approaches, technology usage, and systemic revisions. Clinical, training, and research recommendations and implications are reported. Clinical implications include raising awareness around the lack of neurodivergent‐affirming cancer care. We highlight the need for neuro‐inclusive training for all staff interacting with neurodivergent patients. Such training should be embedded into graduate programs and professional development. We call for co‐designed training (development and delivery) and research, and highlight the lack of current available research. We note the need for empirically driven universal guidelines for neuro‐inclusive oncology care, which we hope will be informed by the present study.

## 1 Background

Approximately 15%–20% of people are neurodivergent, encompassing diagnoses such as autism, attention deficit/hyperactivity disorder (ADHD), intellectual and developmental disabilities (IDD), and learning disabilities [1]. Cancer, which affects approximately 40% of individuals over the lifespan (National Cancer Institute, 2025), is a leading cause of death among autistic people [2], and those with IDD [3].

Neuro‐inclusive cancer care remains insufficiently researched, despite known adverse physical and psychological outcomes [4, 5, 6] and barriers/challenges in navigating the healthcare system, such as poor hospital experiences and reduced healthcare quality [7, 8, 9]. In turn, oncology providers report obstacles in delivering neuro‐inclusive care, such as inadequate time, training, resources, and care fragmentation [10].

Specialist guidelines for neurodivergent‐inclusive care are emerging, such as for anaesthesia [11], medical imaging [12], and primary care [13]. Yet, no oncology‐specific guidelines exist. This is critical given cancer care, with its complex and variable protocols, frequent appointments, and high‐stress environment, presents additional challenges for neurodivergent patients, warranting suitable accommodations [14].

To promote guideline development, we present the first author's lived experience of navigating cancer as a neurodivergent individual and psychologist, exploring encountered barriers and challenges. Her personal and professional experience uniquely positions her to offer insights into this intersection. Based on her experience, and published research, recommendations for neuro‐inclusive care are provided.

## 2 Case Description

Rebecca (Bec) is a 34‐year‐old late‐diagnosed neurodivergent woman, psychologist, and cancer patient. Her professional area of expertise is working with neurodivergent individuals, providing clinical supervision and training to mental health practitioners in neurodiversity‐affirming psychological practice and interventions. Due to undiagnosed Autism and ADHD (diagnosed at 32 years old), throughout her life, Bec experienced mental health difficulties. In 2024, she was diagnosed with stage IV breast cancer, less than two years after learning of her neurodivergence and connecting with this new identity. Throughout the treatment process, the care that Bec received was insufficiently tailored to the needs of neurodivergent individuals, thereby compounding an inherently distressing ordeal.

### 2.1 Barriers and Challenges

#### 2.1.1 Neurodivergent‐Specific

Throughout appointments, Bec's neurodivergence went overlooked, resulting in pressure to comply with social expectations, including eye contact and engaging in small talk to appear cooperative, leaving her exhausted. Her masking and apparent calmness were mistaken for coping.

In consultations, my neurodivergence was not acknowledged unless I raised it, likely because I’m female, have low‐support needs, mask my symptoms, and received late diagnoses (Autism; ADHD), making my neurodivergence invisible. Due to minimal neurodivergent awareness in oncology, professionals equate appearing “normal” with not requiring support. My efforts to appear functional became grounds for support omission or denial. Unlike some neurodivergent people with visible high‐support needs, who have existing supports, I’ve had to engage in effortful self‐advocacy and make my needs seen/known.

In appointments, which were often brief, Bec received large amounts of information, delivered via vague and rushed verbal instructions without time for clarification or information‐processing. Furthermore, for Bec, unexpected calls about scheduling and test results created uncertainty and anxiety. Amplifying this were inconsistent weekly hospital appointments booked at short notice, without scope for accommodating preferences, which impacted her sense of control and routine.

With accommodations unavailable, the unfamiliar, unpredictable, and sensory‐overwhelming hospital environment was dysregulating (e.g., fluorescent lights, loud machines, strong chemical smells, concurrent conversations, high foot‐traffic). Bec found physically navigating the unfamiliar hospital environment overwhelming. This encompassed attending appointments in various locations without directions, parking instructions, or contact numbers. This logistical confusion compounded existing anxiety.

Bec's appointments were frequently scheduled with different unfamiliar oncologists without prior notice. While these diverse providers had access to her notes, it also promoted a sense of discontinuity of care.

I’ve felt exhausted by having to repeatedly share my story and explain what neurodivergence is to professionals. Initially, when I disclosed my neurodivergence and mental health history, I felt like an inconvenience to the treating team due to my “complexity”. This left me afraid to speak up or ask for accommodations. The shame I felt pressured me to mask my distress. I didn’t want to share my story with new clinicians, as I felt that it would be met with further invalidation or not perceived as clinically relevant.

In medical records, Bec's neurodivergence was either omitted or listed as a treating condition.

In my clinical notes, my neurodivergence was listed as a “medical condition” implying that there is something ‘wrong with me’ requiring treatment and change. This deficit‐based language wasn’t neuro‐inclusive or neuro‐accepting care. At other times, it wasn’t noted at all, making it seem unimportant. Both situations were painful to see.

Not only did Bec encounter interpersonal and communication challenges, but medical procedure difficulties and non‐individualised care, too.

I experienced difficulties with physical examinations and needles and felt that there was an assumption from the treating team that I was just meant to do all those things like everyone else. I’m unsure whether it’s de‐sensitisation or a lack of neurodiversity awareness and knowledge, but either way, trauma‐informed care in these situations was lacking. This is extremely important given a person’s vulnerability when exposing their breasts to an unknown person.

#### 2.1.2 Universal Concerns With Heightened Importance for Neurodivergent Individuals

While these are systemic experiences, universal to many undergoing cancer care, for the neurodivergent individual, they may amplify heightened distress.

Bec's mental health team was excluded from communication by her physical health oncology providers.

Updates regarding my prognosis and health were not communicated to my mental health team, which was important, given that it led to my mental health worsening. I was left to disseminate this news that I wished I didn't have to.

Relative to Bec's physical pain, her mental health was overlooked.

Selective attention exists to pain: I’m often asked, “are you in pain?” but I know this refers to physical pain, not psychological. If physical pain warrants consistent monitoring and intervention, shouldn’t psychological pain too?

Bec received life‐changing news by providers in clinical spaces (e.g., oncologist office, hospital bedside) that offered little emotional support, privacy, processing time, or accommodations to her neurodivergence before being expected to make decisions or leave.

I was often required to leave consultations distressed, without immediate support or mental health follow‐up. On one occasion, after receiving life‐changing results, the consultation ended whilst I was visibly upset. I was ushered out the door and required to walk through a waiting room full of patients. Over the following days, I experienced heightened suicidal ideation that the oncology team were unaware of. When I was visibly upset, oncologists either displayed clinical detachment or were awkward and looked like they had no idea what to say/do. They were unable, unwilling, or perhaps untrained to communicate this news and subsequently accept, tolerate, and hold my distress.

At other times, Bec found that life‐changing test results were abruptly conveyed without pre‐emptive conversation or attempts to be discrete in delivery.

### 2.2 Reflections

I would have liked professionals to try and be neuro‐inclusive. I know that the cancer treatments I’m receiving cannot change, yet accommodations to the interpersonal interactions and systemic protocols punctuating them can. As a mental health professional, if my experience has been difficult, I fear for those neurodivergent individuals without these advantages—those who lack the capacity to self‐advocate. Despite challenges, many small and large changes could transform neurodivergent cancer care.

## 3 Recommendations

Informed by the first author's lived experience and supported by research, Table 1 provides recommendations for the oncology workforce (e.g., providers, ancillary staff, hospital management) to deliver neuro‐affirming care).

**TABLE 1:** Recommended accommodations for providing neuro‐inclusive oncological care.

**Concern for care**	**Recommendation**
**Patient communication**	
Neurodivergence is associated with social and communication differences. For example, for some, there may be disinterest/displeasure in small talk or associated anxiety. Positive, effective communication between provider and neurodivergent (ND) individuals is needed as adverse implications of difficult communication exist e.g., patient misunderstanding, diminished rapport, sharing/documenting inaccurate medical data [17, 18].	**Person‐centred care/communication** **Disclosing and documenting ND status:** Embed optional questions relating to communication preferences and needs into standard clinical conversations and questionnaires. Given the broad heterogeneity within neurodiversity, whereby some ND patients will have delayed processing speed, whilst others might require specifically structured guidance and summaries, integration of questions into patient care will empower patients to self‐advocate and receive care that is specifically tailored to their individualised needs. Similarly, this could include implementing standardised documentation of ND by adding a checkbox or an additional field to patient intake forms that capture ND status, and associated communication preferences and accommodations. This could be framed in a similar way to gender identity, where it is not something to change, but as foundational clinical demographic information necessary to provide appropriate care. It is important to highlight that systemic injustices in access to healthcare have emerged for those that disclose their ND status (e.g., [19, 20, 21]), and we therefore recommend all ND questions remain optional. Given this, it will be important for the ND individual and their support network to evaluate the risks and benefits in disclosing their ND status. Clinicians should not assume that patients who skip these questions are neurotypical and should thereby aim to meet the communication needs and preferences of all patients. 2. **Suitable accommodations:** Offer adjustments to meet individualised patients' needs (i.e., dynamic, flexible, and creative approach to consultations) and preferences (e.g., do not force eye contact or sit/stand too close if this causes discomfort). 3. **Adapting communication:** Tailor professional communication styles to reduce distress, while promoting feelings of safety, trust, and compliance—a known barrier with treating ND individuals [11, 14]. 4. **Non‐stigmatising language:** Use non‐stigmatising language during appointments and documentation. Norms for language differ across neurodivergence, and clinicians should use neutral, non‐stigmatising language that is not deficit‐based (e.g., autism rather than ASD; the former is still accurate but not inherently stigmatising/deficit‐focused) and/or ask patients for their language preferences. **Understanding the patient (through direct questioning):** Identify, document, and share (with the care team) patient ND presentations, preferences, (in)effective strategies, and key concerns. This information can be shared with the care team who can subsequently monitor symptoms, allowing for early identification and supportive intervention. **What are the patient's language preferences?** Asking people their preferences around language, as some ND individuals may prefer identity‐first language (e.g., autistic person), person‐first language (e.g., person with autism), and others may have no preferences. This includes how their neurodivergence is discussed in consultations and written in patient files. If a patient reads medical documents that disregard their language preferences, this can be offencive, invalidating, and damage rapport/trust. **What triggers the patient's distress and may lead to shutdown or meltdown?** Triggers may include hyper/hyposensitivities, difficulties with social communication and unexpected change. *Can you help me to understand any (sensory) triggers? What helps you to feel safe?* **How does the patient indicate anxiety, extreme distress, or complete overwhelm?** *When you get anxious, what does that look like and how is this for you internally? What are some of the early warning signs we can look for? If we notice them, what is the approach we should take to help?* **What are the patients' preferred and effective anxiety management approaches?** *Are there tools you find effective at reducing your anxiety?* **What are the patients' sensory interests and preferences?** Understanding these are important for self‐soothing and self‐regulation. **What are the patients' primary concerns today?** *Is there anything specifically that you are concerned about today? How can I best support you today?* These should be discussed and addressed at the start of the consultation. **Set an agenda:** At the start of the consultation, map out the meeting to allow patients to know what to expect. This will assist in staying within the allocated time frame.
Existing patterns of information sharing may not suitably accommodate the ND patient. This includes information generally being shared verbally. It also includes administrative tasks associated with healthcare (e.g., making appointments, following up on medications, etc) being made via the phone, which can be a barrier to care, as some ND individuals dislike phone calls, finding unexpected calls difficult, and will not answer [22, 23, 24]. Some ND individuals are very tech savvy. This can be utilised to enhance patient care; however, it requires understanding the patient's strengths and preferences.	**Learning and information presentation preferences/needs** Alternative forms of communication could be implemented across all aspects of care (i.e., in‐person, telehealth, audio‐only, and text/chat via online hospital portal communication), including contact for administrative tasks and during consultations. Provide information in advance about when appointments/phone calls will take place and what to expect, to allow time for processing and planning. Offer written/emailed summaries (including easy‐read summaries for people with IDD and LD), visuals, handouts, and links to YouTube videos links. Enquire whether providing audio recordings or transcriptions of appointments would be helpful, if these tools are available. Leverage existing tools and websites to support ND patients to: a. Share information about their ND status with their healthcare providers: i. The Autism Healthcare Accommodations Tool; https://researchautism.org/healthcaretoolkit/accommodationsreport/. b. Learn information about neuro‐inclusive oncology care and available supports: i. MacMillian Cancer Support (UK); https://www.macmillan.org.uk/cancer‐information‐and‐support/stories‐and‐media/blogs/neurodiversity‐cancer‐support ii. The Neuro‐Inclusive Oncology Care and Empowerment Program from the Dana Farber Cancer Institute (USA); https://www.dana‐farber.org/patient‐family/support‐services/neuro‐inclusive‐oncology iii. The Push for Inclusive Cancer Care from the American Association for Cancer Research (USA); https://www.cancertodaymag.org/fall‐2024/the‐push‐for‐inclusive‐cancer‐care/ iv. Resources About Cancer for People with Learning Disabilities from Cancer Research UK (UK); https://www.cancerresearchuk.org/about‐cancer/coping/general‐books‐links/for‐people‐learning‐disabilities Offer the opportunity for patients to ask questions post‐consultation according to their communication needs or preferences (e.g., email or send a message to a provider).
Recognising that ND people have different cognitive profiles, communication should aim to meet these needs to maximise clarity.	**Direct, clear, and concise communication** Straightforward communication with a single point per sentence to minimise content misinterpretation. Ensure patient understanding through direct questioning. Allowing time for information‐processing.
ND patients' are burdened by having to repeat their mental health story [25].	**A multidisciplinary team** Effective documenting, communicating, and sharing of patient information with their multidisciplinary team. Managing and coordinating the team's expectations can mitigate challenges [14]. This may include regular team meetings, sharing patient preferences, and including the patient's mental health practitioner (even if they work at a different institution).
Cancer diagnosis and treatment are inherently distressing, and may be more so for the ND individual given that ND and mental health conditions are closely intersected. For example, over 60% of autistic people, including autistic children, have a diagnosed co‐occurring mental health condition [6]. For some ND patients, this may include symptoms of emotional sensitivity and heightened emotional responses.	**Cancer care that is sensitive to mental‐health (i.e., psycho‐oncology)** Clinicians should consider mental health within cancer care in order to provide appropriate support. 1. **At consultation commencement:** Preface difficult news to prepare the patient and set the consultation tone: *I've got some tough information to share about your recent test results.* 2. **At consultation conclusion following the delivery of difficult news:** a. Validate the ND individual's experience/distress. b. Provide education and facilitate referrals for support services (e.g., psychological, financial, peer‐support). i. If patients are not connected and/or aware of their support eligibility, provide resources and education about additional support services they may be eligible for e.g., community case manager, counselling, or in‐home care/support workers. ii. Practitioners should have access to basic handouts or booklets on mental health that can be given to patients e.g., relaxation, meditation, behavioural activation, and grief/loss. iii. Share news with the care team (incl. Psychiatrist). iv. If needed, liaise with the patient's psychiatrist. 3. **Encourage:** a. Self‐care activities: Ask patients about activities they enjoy and find relaxing and encourage participation and continuation of these activities now and throughout their cancer treatment. b. Drawing upon their support network: *I'd encourage you to connect with personal and professional support.* 4. **Check‐in:** *Please take care of yourself, this has been a difficult appointment, how is the news sitting with you? Are you getting the support that you need?* In follow up consultation(s), review the patient's mental health in the context of treatment/and diagnosis. This could include asking about psychological distress and screening of anxiety/depression, where applicable.
Certain ND populations (e.g., autism) are more likely to experience trauma and adverse life experiences, so care should be trauma‐informed [26].	**Trauma‐informed care** Meeting the client on a human level with gentle supportive care to create safety. Factoring in additional time for potential emotional responses. Avoiding practices that can lead to re‐traumatisation (e.g., respect sensory needs; provide concrete advanced explanations, honour ‘stop’ signals, allow recovery time, offer procedural choices). Providing patients with choice and autonomy in their cancer care, focus on empowerment and involvement in decision making.
The healthcare system is not designed for neurodivergence, and can create sensory overload, communication barriers, and power imbalances, potentially leading to adverse outcomes such as poor health and care avoidance.	**Encouraging social supports** Support people can assist with promoting feelings of safety, comfort, and emotion regulation. They can also facilitate communication, collaboration, patient advocacy, and may support the patient with information‐processing. Whether social support be in the form of a hospital‐provided patient advocate or a part of an existing support network (e.g., family/friend), ND people should be able to and encouraged to bring along advocates/support people wherever possible. Consideration of supporting people's input and insights should be taken into account.
	**Offering ND patients with a hospital‐provided patient advocate** 1. ND people should be provided with the option of having a healthcare advocate included within their care team. This may be important as autistic people, for example, have on average smaller social and support networks than others. 2. If the ND patient wishes, this patient advocate should be invited to all appointments, procedures etc.
**Physical environments**	
Hospitals can be sensory dysregulating environments with bright lights, noise, many people etc.	**Accommodations to physical environments** **N** **on‐/clinical space adaptations:** Offer calm environments and remove unnecessary sensory stimuli [27]. In clinical and non‐clinical spaces, where possible, adjustments can be made to noise, smell, temperature, lighting, and number of people. For example: Access to quiet waiting rooms or retreat spaces before/after sensory‐demanding medical interventions (e.g., MRI) or after receiving difficult treatment news. Large chemotherapy rooms with many patients could include a quiet zone with privacy curtains, dimmable lights, reduced foot traffic, and sensory kits. **Access to sensory support items:** This could include the availability of fidget toys, soft pillows, weighted blankets, music, noise cancelling headphones, and ear plugs. Sensory coping kits can also be offered, or patients can be encouraged to bring their own. It could also include the use of virtual reality, phone, and tablet use for procedure distraction and pain management. **Food and drink:** Due to sensory issues and gastrointestinal conditions that require dietary restrictions [28], many ND individuals struggle with food options provided in hospitals. Discuss with patients and offer options to accommodate dietary needs/preferences, encouraging them to bring ‘safe foods’ to hospital if unavailable. Note in the patient file if there are particular foods and drinks that the patient *does* like and that can be offered. Inadequate food and drink consumption may increase the likelihood of emotional dysregulation. **ND‐inclusive signage and symbols:** Clear signage and use of visual displays. This can include the rainbow infinity symbol to represent ND, and/or the sunflower symbol to represent non‐visible disabilities more generally to denote ND inclusion and safety. **Draw upon existing frameworks to facilitate equitable clinical services for ND individuals:** E.g., The Autistic SPACE Framework [29].
Unfamiliarity and changes to routine can be dysregulating for some ND individuals.	**Patients' familiarisation with the clinical team, physical spaces, and treatments** Providing orientation information to ND patients [30] before consultations, tests, or treatments may ease anxiety, so the patient has a degree of familiarity of what they are looking for on their first appointment. For example: Brief pre‐recorded videos of clinicians professionally introducing themselves, and a non‐work interest (e.g., hiking) to promote rapport development. Pre‐recorded videos of medical imaging machines alongside a brief description of what a procedure might entail. Sharing approximate duration of consultations, procedures, and treatments. Photos of the outside of the buildings and treatment rooms. Building details (e.g., leaflets with parking instructions, direct phone numbers).
**Physical pain**	
Understanding that ND people can experience physical pain differently [31]. This includes high pain thresholds and the presentation of mismatched affect in situations where outward expression of pain would be assumed.	**Physical pain management considerations and accommodations** Providers to communicate understanding and awareness of ND pain differences and potential challenges with physical examinations and needles. Validate and acknowledge difficulties: *I'm sorry you are going through this. We will be here alongside you to make the journey as comfortable as possible.* Practical accommodations for needle‐based procedures (e.g., options for port implantations for medication delivery; use of numbing creams; option to lay down if prone of fainting; ultrasound for cannulation if locating veins difficult; offer breaks). Enquire about the patients' pain and offer appropriate pain management (i.e., PRN medications) based on their report of pain, even if they do not look in discomfort. Conversations around appropriate medications should be discussed at the commencement of treatment, to ensure the patient has appropriate access to the medications they may require, and they are listed on their medical chart. Discuss pain‐management strategies (e.g., distraction through sensory objects, bringing and talking to a support person, listening to music, preference to sit silently and not engage socially). Acknowledge and celebrate the completion of difficult procedures and the patients' progress in distress tolerance.
**Administrative approaches**	
Hospitals' standard brief and efficient consultations may not accommodate ND patients' needs. In particular, inconsistent hospital staff can negatively impact the ND patient, resulting in frustration, reduced trust, and rapport.	**Extended appointment times:** Offering longer appointments to enable information‐processing and rapport building. **Appointment continuity:** Where possible, schedule regular appointments on the same day and at the same time each week/month to create predictability. Patients can be asked if they have a preferred appointment time and day. **Scheduling appointments:** Provide appointment scheduling options and allow ND people to make informed choices about when to schedule. Example scheduling options include: 1. Determine with patients which times of day and which locations are most suitable, as this can have a notable impact on the accessibility of appointments. Whilst it may not always be possible to accommodate patient preferences, efforts to accommodate these aspects of neurodiversity will ultimately promote engagement, punctuality, and cooperation whilst minimising distress and shutdown. 2. Discuss with patients the balance between receiving timely care and accommodations directly with patients, particularly in the context of time‐sensitive treatments, such as chemotherapy, radiation, and surgery. Care should be taken to provide clear and accurate information, offer flexibility in scheduling for appointments or treatments that are not urgent, and acknowledge the distress that appointments or treatments may cause. **Continuity of care and a treatment team** Allowing patients to see the same providers, wherever possible. Maximise patient familiarity, predictability, comfort, and safety.
Extensive medication regimes may be tedious, confusing, and difficult to remember.	Medication organisation: Suggest use of weekly pill organisers, which can be prepared by a pharmacist. Delegation of medication management: Encourage a support person to take on the role of managing medications and staying up to date with relevant changes. Offer practical strategies to promote medication compliance: E.g., use of phone alarm reminders, mobile applications, or pairing medication administration with well‐established routines (after meals or brushing teeth).
**Systemic revisions**	
A lack of knowledge, understanding, language, and expertise among healthcare professionals or dated knowledge of ND conditions [10, 25].	**Education and training** Training, not only for professional staff, but ancillary staff liaising with patients. Preferably training and education would be part of ongoing professional development. Training needs to include research, theoretical, and clinical elements. Training content development and delivery would ideally include those with a lived experience. Critically, training also needs to consider that prevalence rates are often in flux and vary according to new understanding and shifts in diagnostic criteria. Education and training programs should include the following: **Research** **Understanding up‐to‐date ND prevalence rates, current information provided below:** 15%–20% of all people identify as ND [1]. ∼3% of children are autistic, and autism is 3.4 times more common in boys than in girls [32]; 6% have ADHD [33]; 9% of children have a LD [34]; 1% have an IDD [35]. ADHD and autistic people have a reduced life expectancy (O'Nions et al., 2024; [36]). As described above, many ND people remain undiagnosed which significantly impacts their health, education, and employment (French et al., 2022). **Understanding co‐occurring conditions:** The co‐occurrence of ND conditions (e.g., ∼28% of individuals will have autism *and* ADHD; [37]). Co‐occurring mental health conditions are more common in those who are late‐diagnosed autistic, compared to those who are early‐diagnosed [38]. Co‐occurrence of ND conditions *and* mental health conditions (e.g., > 60% of autistic people have a diagnosed co‐occurring mental health condition [6]). Co‐occurrence of ND conditions *and* physical illnesses (e.g., Autistic people are 46% more likely to have heart disease [39]). **Theoretical** **Neurodiversity acceptance:** Society has often sent the message that there is something wrong with ND individuals and that they need to be ‘fixed’ [40]. However, ND is often not perceived as a mental health condition, disease, or even a mental health term. Instead, ND is perceived as differences in thinking/cognition, not pathology [13]. **Contextual information and historical treatment:** ND individuals have higher rates of trauma than the general population [26]. For example, some ND individuals describe early childhood life experiences and medical interventions as ‘traumatic’. There has also been historical mistreatment of neurodivergent people, including being institutionalised. Given this, they may be wary of health professionals and new people, due to prior experiences of bullying, discrimination, or mistreatment. Such knowledge can contextualise to the provider why it may be more difficult to develop rapport and help them to feel safe. **Familiarity with ND diagnostic revisions:** (i.e., retirement of Aspergers Syndrome; introduction of ASD, levels 1–3). **Late ND diagnoses:** Many people, particularly women, received late ND diagnoses. These are equally valid as someone with more overt ND symptoms diagnosed in childhood. Receiving such a diagnosis at a later age can be an important part of understanding oneself and may lead to experiencing varied emotions, whilst also shaping a sense of personal identity. Different individuals will respond in their own way to their late diagnosis—for some, it will provide a sense of relief and celebration. For others, it may be a difficult thing for them to come to terms with (e.g. grief, anger). Clinicians should not assume that a diagnosis of autism and/or ADHD is upsetting or viewed negatively. **Language around neurodiversity** [41]: The use of language has negative consequences for the ND individual, including marginalisation and dehumanisation (e.g., [42]). Language preferences should relate to how the patient is discussed in consultations *and* what is written in patient files. If a patient reads medical documents where they have been referred to in a way they consider offencive this may be invalidating and harmful. Therefore, the choice of language and inclusion of specific details have a notable impact‐both during healthcare appointments and in written notes. Language norms are different across neurodivergence, for example person‐first language is still preferred for IDD/LD, but identity‐first language is preferred for autism. Given this, and as mentioned above, we advocate for clinicians using neutral language (e.g., Autism instead of ASD); this can help build rapport and terms like ‘disorder’ may unnecessarily stigmatise people [43]. Best practice is to ask the patient their preferences. Understanding the difference between identity‐first versus person‐first language. Heterogeneity within neurodiversity: This may encompass the inclusion of varied diagnoses; diverse symptom profiles across genders; high/low support needs; and diverse symptom presentations (e.g., some may mask, appearing fine externally—dyssynchrony between internal affect and external presentation) [44]. **Clinical:** Practical guidance on how to engage in effective neuro‐inclusive communication [45]. Understanding medication interactions between medications and risks—stimulants and anxiety medications and cancer drugs—cost‐benefit holding. Neurodiversity differences in physical pain [31]. **Leverage existing websites to learn about how to best support ND patients:** a. Clinical Care for Autistic Adults from Harvard University (USA); https://pll.harvard.edu/course/clinical‐care‐autistic‐adults b. Oncology news Central; https://www.oncologynewscentral.com/oncology/oncologists‐and‐neurodivergent‐patients‐how‐to‐identify‐issues‐and‐improve‐care
Currently, no ND‐specific resources exist to guide cancer care.	**Develop evidence‐based ND‐specific oncology clinical implementation tools and training toolkits** These may include: Pre‐consultation checklists (e.g., sensory preferences, communication preferences) During consultation guidelines (e.g., language prompts, visual aids). Post‐consultation resources (e.g., consultation summary templates, follow‐up accommodation plans) Practitioner ND oncology training toolkits These should be codesigned with ND cancer patients, ND cancer survivors, and their families, and made freely accessible to healthcare teams.
ND accommodations should not be static, but open to patient feedback and evolving knowledge.	**Receptivity to feedback** Openness to change, adjustments, and learning based on: ND patient feedback—physical/online anonymous feedback boxes. New research findings and changing standards/practices. Ongoing discussion with patient and care team, to seek feedback on current approach being utilised. As a provider, invite patients to let you know if you have said/done something in a way they do or do not like.
Trauma is not exclusively an intrapersonal or interpersonal issue [46], but can occur at the organisational/structural/collective level rooted in oppression. Developing trauma‐informed organisations will likely reduce the risk of ND re‐traumatisation and enhance patient engagement and outcomes.	**Developing a trauma‐informed organisation and preventing re‐traumatisation** **[** 46, 47]. **Trauma‐informed organisations:** Embed trauma‐informed policies, practices, and frameworks (e.g., establish an organisational commitment to trauma‐informed care; train and equip *all* staff to provide supportive and effective services; appreciate how trauma influences ND behaviour; avoid pathologising; conduct universal trauma screenings; recognise that ND individuals carry unspoken trauma histories that influence their health and healthcare engagement). **Trauma‐informed leadership:** This may include offering trauma‐informed supervision; hiring trauma‐informed staff; developing a climate of safety including transparency; choice; diversity awareness; and collaboration.

Abbreviations: AI, Artificial Intelligence; ASD, Autistic Spectrum Disorder; IDD, Intellectual and Developmental Disabilities; LD, Learning Disability; MRI, Magnetic Resonance Imaging; ND, Neurodivergent.

Many of these recommendations would likely improve the quality of care for all people—not just neurodivergent people, though they will have an outsized impact on quality of care for those who are neurodivergent. In addition, applying these recommendations broadly serves to improve the quality of care for undiagnosed neurodivergent people, as many neurodivergent people are still unrecognised today, and is associated with negative outcomes [15, 16].

Given the broad heterogeneity with which neurodiversity presents, application of these recommendations will require flexibility and careful tailoring from clinicians, contingent on the unique circumstances of the patient in question.

## 4 Implications

Bec's experiences have implications for training, practice, and research. Neuro‐inclusive oncology care and practice should be integrated into graduate programs and form part of ongoing professional development, with such professional training available not only to medical staff, but anyone interacting with ND patients. Naturally, any such training material should be co‐designed with neurodivergent individuals.

With respect to practice, we hope that Bec's story will raise awareness around the current lack of neurodivergent‐affirming oncology care, and that our recommendations are integrated into routine clinical work. In relation to research, we highlight the dearth of literature around neuro‐inclusive oncology, highlighting the need for qualitative and quantitative research to explore the experiences of ND individuals' cancer care. Ideally, such future research should not only include ND participants but be co‐designed alongside ND individuals. Without hearing from people with lived experience, it is unlikely that existing barriers and challenges to neuro‐inclusive care will be meaningfully addressed. We hope the present case study highlights the need for empirically driven universal guidelines for neuro‐inclusive oncology care.

## 5 Key Takeaways

– ND patients face systemic and interpersonal barriers in cancer care, with lived experience highlighting urgent gaps and risks.
– There is an urgent need for trauma‐informed, collaborative care, and better integration of mental health support.
– Neuro‐inclusive oncology is an understudied area and further research is required.
– The development of formal evidence‐based training and guidelines for healthcare practitioners is required to provide neuro‐inclusive oncology.
– We recommend six key domains for improvements in neuro‐affirming care: Patient communication, physical environments, physical pain, administrative approaches, technology usage, and systemic revisions.

## 6 Conclusion

Cancer treatment is an inherently distressing process. For neurodiverse patients, the absence of neurodiversity‐affirming guidelines means that this distress will be markedly compounded, causing unnecessary further psychological harm to individuals who already have higher rates of trauma. Neurodiversity‐affirming care will likely reduce trauma and distress in an experience (i.e., cancer care) that is already traumatic for many, resulting in better quality of life for ND patients whilst receiving cancer treatment. Implementing these recommendations would likely make a notable difference on ND patients throughout their cancer journey and emphasise the importance of providing such care, which will begin to challenge years of exclusion, discrimination, and ableism. If neurodivergent‐inclusive approaches are applied, it is likely that ND individuals will seek medical care earlier, receive all necessary treatments, thereby reducing mortality rates and healthcare costs in this population. Clinicians and services could easily implement many of these recommendations. Other recommendations will require higher‐level system changes before down‐stream application and benefits can be realised.

## Author Contributions

**Rebecca Trevethick:** conceptualisation, investigation, methodology, resources, writing – original draft, writing – review and editing. **Simon Baron‐Cohen:** investigation, resources, supervision, writing – original draft, writing – review and editing. **Elizabeth Weir:** investigation, resources, writing – original draft, writing – review and editing. **Carrie Allison:** investigation, resources, writing – original draft, writing – review and editing. **Matthew C. Fysh:** investigation, resources, writing – original draft, writing – review and editing. **David P. Laplante:** investigation, resources, writing – original draft, writing – review and editing. **Malini Dey:** investigation, resources, writing – original draft, writing – review and editing. **Jessica E. Opie:** conceptualisation, investigation, methodology, project administration, resources, supervision, writing – original draft, writing – review and editing.

## Funding

SBC received funding from the Wellcome Trust 214322\Z\18\Z. For the purpose of Open Access, the author has applied a CC BY public copyright licence to any Author Accepted Manuscript version arising from this submission. SBC also received funding from the Innovative Medicines Initiative 2 Joint Undertaking under grant agreement No 777394 for the project AIMS‐2‐TRIALS. This Joint Undertaking receives support from the European Union's Horizon 2020 research and innovation programme and EFPIA and AUTISM SPEAKS, Autistica, SFARI. SBC also received funding from Autism Action, SFARI, the Templeton World Charitable Fund and the MRC. The funders had no role in the design of the study; in the collection, analyses, or interpretation of data; in the writing of the manuscript, or in the decision to publish the results. Any views expressed are those of the author(s) and not necessarily those of the funders (including IHI‐JU2). All research at the Department of Psychiatry in the University of Cambridge is supported by the NIHR Cambridge Biomedical Research Centre (NIHR203312) and the NIHR Applied Research Collaboration East of England. The views expressed are those of the author(s) and not necessarily those of the NIHR or the Department of Health and Social Care.

## Ethics Statement

The authors have nothing to report.

## Conflicts of Interest

The authors declare no conflicts of interest.

## Data Availability

Data sharing is not applicable to this article as no datasets were generated or analysed.
